# tRNADB-CE: tRNA gene database well-timed in the era of big sequence data

**DOI:** 10.3389/fgene.2014.00114

**Published:** 2014-05-01

**Authors:** Takashi Abe, Hachiro Inokuchi, Yuko Yamada, Akira Muto, Yuki Iwasaki, Toshimichi Ikemura

**Affiliations:** ^1^Graduate School of Science and Technology, Niigata UniversityNiigata, Japan; ^2^Nagahama Institute of Bio-Science and Technology, NagahamaShiga, Japan; ^3^Faculty of Agriculture and Life Science, Hirosaki UniversityHirosaki, Japan

**Keywords:** tRNA, database, metagenome, phylogenic maker, BLSOM, big data

## Abstract

The tRNA gene data base curated by experts “tRNADB-CE” (http://trna.ie.niigata-u.ac.jp) was constructed by analyzing 1,966 complete and 5,272 draft genomes of prokaryotes, 171 viruses’, 121 chloroplasts’, and 12 eukaryotes’ genomes plus fragment sequences obtained by metagenome studies of environmental samples. 595,115 tRNA genes in total, and thus two times of genes compiled previously, have been registered, for which sequence, clover-leaf structure, and results of sequence-similarity and oligonucleotide-pattern searches can be browsed. To provide collective knowledge with help from experts in tRNA researches, we added a column for enregistering comments to each tRNA. By grouping bacterial tRNAs with an identical sequence, we have found high phylogenetic preservation of tRNA sequences, especially at the phylum level. Since many species-unknown tRNAs from metagenomic sequences have sequences identical to those found in species-known prokaryotes, the identical sequence group (ISG) can provide phylogenetic markers to investigate the microbial community in an environmental ecosystem. This strategy can be applied to a huge amount of short sequences obtained from next-generation sequencers, as showing that tRNADB-CE is a well-timed database in the era of big sequence data. It is also discussed that batch-learning self-organizing-map with oligonucleotide composition is useful for efficient knowledge discovery from big sequence data.

## INTRODUCTION

After completion of genome sequencing, tRNA genes on each genome have been predicted using computer programs such as tRNAscan-SE (Lowe and Eddy, 1997), ARAGORN (Laslett and Canback, 2004), and tRNAfinder (Kinouchi and Kurokawa, 2006) and included in the flat file of the sequence data for registration to the International DNA Data Banks (DDBJ/ENA/GenBank). However, in approximately 5% of the completely sequenced genomes, the annotation data of tRNA genes have not been included. There also exist many cases where an important information of anticodon (and thus of amino acid) has not be added while the tRNA itself is noted. The compilation of tRNA sequences and tRNA genes has been published for the first time by Sprinzl et al. (1978) and has been updated and reconstructed the database including modified nucleosides (tRNAdb^1^; Jühling et al., 2009). Using tRNAscan-SE, the Genomic tRNA Database (GtRNAdb^2^) has been constructed for complete and near complete genomes (Chan and Lowe, 2009). In addition, a database for comprehensive listing of modified nucleotide (The RNA Modification Database^3^; Limbach et al., 1994) and that for modification pathways (MODOMICS^4^; Machnicka et al., 2012) have been reported. Mamit-tRNA^5^ is a compilation of mammalian mitochondrial tRNA genes (Pütz et al., 2007). Another characteristic tRNA database is SPLITSdb^6^ constructed by Keio Group (Sugahara et al., 2006, 2008), which has compiled tRNA sequences in archaeal and primitive eukaryotic species for promoting next studies of tRNA evolution and processing; 671 intron-containing and 12 split tRNAs have been registered. In the present Review Article, we introduce current status of our “tRNA gene database curated by experts (tRNADB-CE)” and compare it with other tRNA databases for explaining its characteristics.

In accord with the remarkable progress of DNA sequencing technology, a vast quantity of partially sequenced draft genome sequences and metagenomic sequences obtained from a wide variety of environmental and clinical samples have been compiled in DDBJ/ENA/GenBank. However, no information of tRNA genes has been added to draft and metagenomic sequences in DDBJ/ENA/GenBank and in GtRNAdb and tRNAdb. Metagenomic sequences have attracted broad industrious interests; even short sequences obtained with new-generation sequencers contain a large number of full-length tRNAs because tRNA lengths are short. Search for tRNA genes in metagenomic sequences may provide a new strategy for clarifying the microbial community in an environmental sample. This prediction is supported by the findings that examination of a group of tRNAs with an identical sequence obtained from species-known prokaryotes has revealed that such tRNAs belong primarily to a specific phylogenetic group and that the phylotype-specific tRNA sequences have also been found in species-unknown metagenomic sequences. This supports the view that these tRNA genes should become good phylogenetic markers for studying phylotype composition in an environmental ecosystem. It should be noted here that, when analyzing a dataset composed only of short fragmental sequences (e.g., 100 nt), genomic sequences other than tRNA genes appears to be difficult to be properly used for phylogenetic assignments, except for dominant genomes for which contiguous sequences with a sufficient length for constructing reliable phylogenetic trees can be obtained.

## GENOMIC SEQUENCES ANALYZED AND METHODS OF SEARCH FOR tRNA GENES

Because one important role of tRNADB-CE is a use of tRNA genes as phylogenetic markers in metagenomic studies, we have mainly focused on microbial genomes. The following sources of DNA sequences have been used for constructing the present version of tRNADB-CE: 1966 and 171 complete prokaryote and virus genomes released by DDBJ/EMBL/GenBank up to September 2012, 121 complete chloroplast genomes released by organelle genome database (GOBASE^7^) up to March 2009, 5272 prokaryote draft genomes released by WGS division in DDBJ/ENA/GenBank up to September 2012, 12 eukaryote complete genomes, 17 million metagenomic sequences released by DDBJ/ENA/GenBank up to March 2012, and 217 million metagenomic sequences obtained using next-generation sequencers and released by sequence read achieve (SRA^8^) in NCBI up to March 2012.

To enhance the completeness and accuracy of searching for tRNA genes, three computer programs, tRNAscan-SE, ARAGORN, and tRNAfinder have been used in combination, since their algorithms are partially different and have rendered somewhat different results. The tRNA genes found concordantly by all three programs were stored in tRNADB-CE after brief manual checking for cases of non-standard anticodons, such as bacterial A-starting anticodons (except for Arg) and those responding to termination codons, as explained later. Discordant cases among the three programs (approximately 5% of the total of tRNA gene candidates predicted by at least one program) were manually checked by three experts (YY, AM, and HI) in tRNA experimental fields and were classified into three categories: (A) reliable tRNA genes, (B) not tRNA genes, and (C) ambiguous cases.

The tRNA genes of Archaea obtained from SPLITSdb^9^ constructed by Keio Group (Sugahara et al., 2006, 2008) are included in the current version of tRNADB-CE. Basic functions of the database have been described previously (Abe et al., 2009, 2011). For fragment sequences obtained by metagenome studies, only tRNA genes found concordantly by the three programs and those with sequences identical to the tRNAs already included in the database were stored. Many tRNA genes were detected in various environmental samples, and the number was registered for each environment sample. This result has enabled us to predict the microbial population in an environmental ecosystem by using tRNAs as phylogenetic markers. Since a significant portion of environmental sequences is thought to be derived from unculturable microbes, tRNA genes of novel microbes should be included.

## tRNA GENES COMPILED IN THE CURRENT VERSION AND A NEW FUNCTION FOR ORGANIZING COLLECTIVE KNOWLEDGE

**FIGURE 1:**
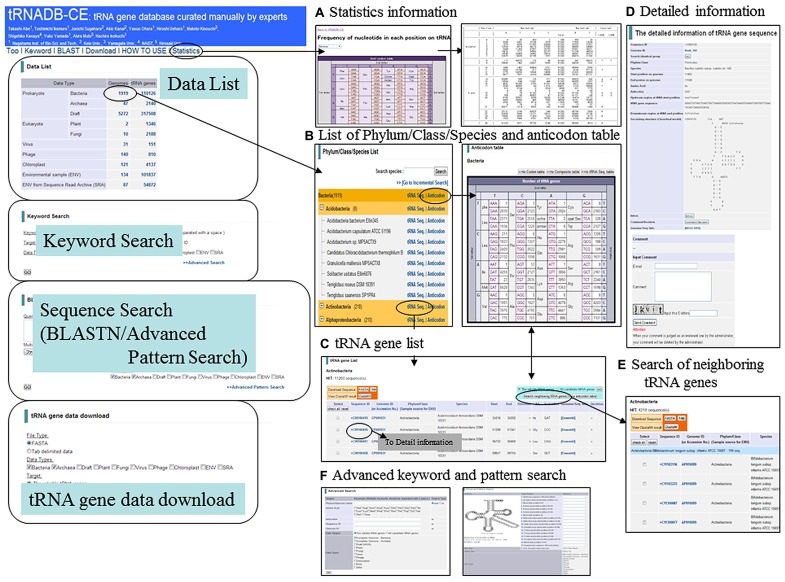
**Basic functions of tRNADB-CE. (A)** Statistics information for frequency of nucleotide in each position on tRNA cloverleaf secondary structure, **(B)** List of Phylum/Class/Species and anticodon table, **(C)** tRNA gene list, **(D)** Detailed information, **(E)** Search of neighboring tRNA genes, and **(F)** Advanced keyword and pattern search.

In the present database, 595,115 tRNA genes in total (112266, 317508, 961, 3534, 4137, and 156709 genes from 1966 complete and 5272 draft prokaryote genomes, 171 viruses, 121 chloroplasts, 12 eukaryotes, and 221 metagenomic samples, respectively) have been registered. This number is two times as many genes as were previously registered (Abe et al., 2011) and functions of the database are listed in **Figure 1**. Comparison of registered data and functions among three tRNA databases is presented in **Table 1**. More than ten and five times of tRNAs have been compiled by tRNADB-CE than by tRNAdb and GtRNAdb, respectively. This is mainly because tRNADB-CE has included tRNAs obtained from draft genome and metagenome sequences, as well as virus and plastid sequences. Another important difference of tRNADB-CE from others is the use of three computer programs for tRNA gene search.

**Table 1:** Comparison of registered data and functions between databases (at March, 2014).

	tRNAdb	GtRNAdb	tRNADB-CE
**No. of tRNA sequences**	**42571**	**111385**	**595115**

**No. of genomes and samples**			
Eukaryotes	287	82	12
Prokaryotes	292	915	1966
Draft genomes (prokaryotes)	None	None	5272
Metagenome samples	None	None	221
Viruses	8	None	171
Plastids	53	None	121
Mitochondria	1418	None	None
**tRNA finding methods and sources**			
Sequence sources	Published tRNA seq., genomic seq.	Genomic seq.	Genomic seq.
Prediction programs	tRNAscan-SE	tRNAscan-SE	tRNAscan-SE, ARAGORN, tRNAfinder
Manual curation	°	None	°
**Viewable data**			
Anticodon usage	None	°	°
Sencondary structure	°	°	°
Alignment result	°	°	°
Nucleotide modification	°	None	None
**Search method**			
Species and phylogenetic names	°	°	°
Anticodon/amino acid	°	°	°
Sequence homology (BLAST)	°	°	°
Oligonucleotide pattern	°	None	°

The nucleotide frequency in each position on the tRNA cloverleaf structure is presented on a statistics page, providing information on consensus nucleotides for each anticodon of individual data types (**Figure 1A**). Since a vast amount of tRNAs has been registered, this is useful for systematic search for identifier nucleotides, which interact with aminoacyl-tRNA synthetases. To aim at creating a high quality database by collecting knowledge in various tRNA research fields, we developed a new function for including comments on each gene in “The detailed information of tRNA gene sequence page” (**Figure 1D**). Users can add comments after typing in their e-mail address and password, while we reserve the right to remove irrelevant comments. We hope that the accumulation of user’s comments will provide annotations with high quality and this database will become an information sharing system in the tRNA research community.

## IDENTICAL SEQUENCE GROUPS AND THEIR USE AS PHYLOGENETIC MARKERS

We investigated a group of tRNAs with an identical sequence and found that one group was composed primarily of tRNAs derived from species belonging to a certain phylogenetic group, i.e., phylotype-specific tRNAs. To verify this phylogenetic preservation of tRNA sequences in more detail, we conducted the clustering of 429,774 tRNA sequences derived from 7,237 prokaryote genomes through CD-HIT sequence alignment (Li and Godzik, 2006). Then we designated the group with an identical sequence as “Identical Sequence Group: ISG”. When focusing on ISGs composed of more than five genes, 95% of ISGs have been conserved at the phylum level, showing that most tRNA sequences are good phylogenetic markers at least at the phylum level. In addition, approximately 65% of ISGs have been conserved at the genus level, indicating that more than half of ISGs may be usable as genus-specific makers. This is because tRNA sequences have been stably conserved during evolution. Increases in the number of genes in each ISG in the near future will progressively clarify the phylogenetic range that can be covered by one ISG. By combining the data provided by this database with other knowledge obtained from experiments or literatures, users can choose useful phylogenetic markers (e.g., genus-specific markers) by themselves. Our group has started to search phylogenetic markers for the genomes that are rare in regular environments and will publish these markers in the aforementioned column for comments to each tRNA gene. It should be noted here that horizontal gene transfer between different species is a general characteristic of microbial genomes. Therefore, we may not find phylogenetic markers with the 100% accuracy. Any informatics method, including sequence homology searches, most likely assigns the horizontally transferred elements to the donor but not the recipient genome. When certain tRNAs that have primarily found in a certain phylotype have been additionally found in the phylogenetically distant but restricted species, these tRNAs may represent genes that have been transferred horizontally to the restricted species or products of the convergent evolution. Phylogenetic marker tRNAs have to be used in consideration of these points.

**FIGURE 2:**
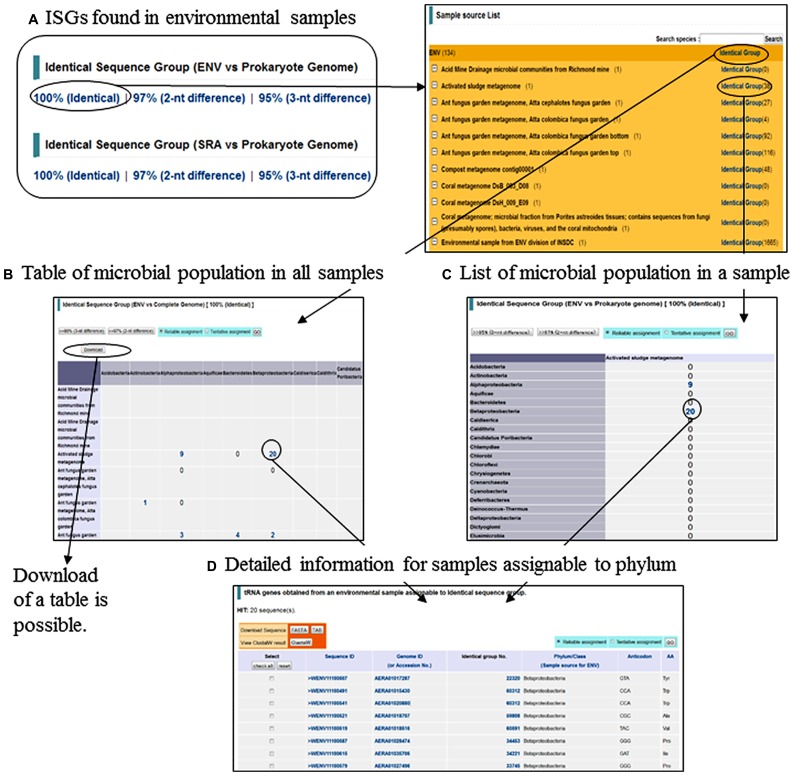
**List and search for (A) Identical sequence groups (ISGs) found in environmental samples, (B) Table of microbial population in all samples, (C) List of microbial population in a sample, and (D) Detailed information for samples assignable to phylum**.

Interestingly, approximately 25% of tRNA genes obtained from metagenomic sequences were found identical in sequence to genes from species-known prokaryotes and assignable to ISGs. By using these assigned tRNAs as phylogenetic markers, we have predicted the microbial community in an environmental ecosystem at a phylum level (**Figure 2A**). The number of tRNA genes found in known species and a list of the species can be browsed for each environmental sample (**Figures 2B,C**). When the numbers are clicked, the list of metagenomic tRNAs assignable to phylum can be accessed (**Figure 2D**). This strategy can be applied even to the data of short sequences obtained with new-generation sequencers, such as metagenomic sequences in the SRA^10^ at NCBI. It is noteworthy that phylogenetic clustering of short sequences (e.g., 100 nt) with conventional sequence homology searches is inadequate for studying a microbial community in an ecosystem. However, evolutionary stable tRNA genes can be used as effective phylogenetic markers for predicting the microbial community, since full-length tRNAs can be found even from short genomic fragments obtained with new-generation sequencers.

The database is also capable of searching for sequences with 1–3 nt differences (**Figure 2A**). By using tools in the database as well as phylogenetic markers found by users (e.g., genus-specific markers), users can search for very specific and rare genomes of the user’s attention from a wide range of environmental samples.

## MINIMUM ANTICODON SET AND NON-STANDARD-TYPE tRNAs

An important curation process by experts was to investigate the minimum anticodon set most likely to be essential for the translation system of each bacterial species (Osawa, 1995), because three computer programs did not necessarily assign this set concordantly. When we could not find the minimum set in our standard search, we reexamined the candidates predicted by one or two programs and searched for the most probable candidate that is satisfactory to the minimum set, according to various criteria such as identifiers for respective anticodons and referring to literatures of experiments. We also checked whether an identical sequence and the sequences with 1–2 nt differences were present in closely related genomes, basing on the view that a functionally active gene should be stably maintained during evolution. This final check has become increasingly useful because many closely related species have been sequenced currently. The search for the minimum anticodon set in each genome can assign tRNAs whose sequences appear to be rather non-standard. If non-standard-type tRNAs have been found iteratively in species belonging to a special phylogenetic group, such tRNAs will become good phylogenetic markers with high specificity. The aforementioned newly added column for comments on each tRNA can be used for mentioning this phylogenetic information.

**FIGURE 3:**
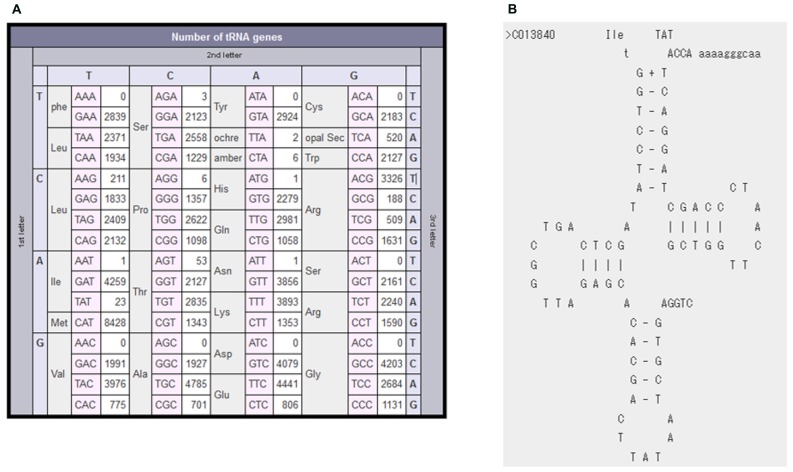
**Examples of non-standard-type tRNAs**. **(A)** The anticodon occurrence table of bacterial tRNAs. It is clear that A-starting anticodons are very rare in bacterial tRNAs. **(B)** Cloverleaf secondary structure of *M. mobile* tRNA^Ile2^.

We next explain one example of non-standard-type tRNAs more in detail. The Anticodon Table of bacterial tRNAs (**Figure 3A**) points out that occurrences of A-starting anticodons are very rare except for Arg, Leu, and Thr: 0 for Phe, Tyr, Cys, Val, Asp, and Gly; 1 for His, Ile, and Asn; 3 for Ser and 6 for Pro. We have manually checked tRNA candidates with these very rare A-starting anticodons, on the basis of the aforementioned criteria for the minimum anticodon set and/or the iterative occurrences in the closely related species. Information concerning such non-standard-type tRNAs can promote experimental studies to prove predicted tRNA candidates at the RNA level. For example, we have found novel bacterial tRNA genes with TAT anticodon in 19 species, including *Mycoplasma mobile*. In many bacteria, ATA codons are deciphered by tRNA^Ile2^ bearing lysidine (L) at the wobble position (Soma et al., 2003; Suzuki and Miyauchi, 2010); L is a modified cytidine introduced post-transcriptionally by tRNAIle-lysidine synthetase (TilS). Some bacteria, including *M. mobile*, do not carry the tilS gene, indicating that they have a specific system to decode ATA codons. Taniguchi et al. (2013) have experimentally shown at the cellular RNA level that the *M. mobile* tRNA^Ile2^ registered in our database (**Figure 3B**) contains an unmodified UAU anticodon.

## tRNA DATABASE WELL-TIMED IN THE ERA OF BIG SEQUENCE DATA

One important characteristic of tRNADB-CE is inclusion of tRNAs found in a large number of partially sequenced genomes. The aforementioned search for the minimum anticodon set can be applied only to a complete genome. Another check that is applicable even to partially sequenced genomes is to examine whether non-standard-type candidates have been repeatedly found in closely related species, and this process has become increasingly useful because genomes of many closely related species, even of different strains belonging to one species, have been sequenced. When the same or almost the same sequence has been found repeatedly, we have included the sequence in the Reliable category basing on the knowledge that functionally important genes have been stably maintained throughout evolution. Importantly this check process can be applied even to metagenomic sequences; if the non-standard-type candidates have been found repeatedly in metagenomic samples, especially in different environmental samples, these can be included in the Reliable category. Accumulation of a massive number of sequences in the near future should further increase the usefulness and reliability of this strategy, indicating that tRNADB-CE is a well-timed database in the era of big sequence data.

## ENHANCING QUALITY OF A LARGE-SCALE DATABASE AND EFFICIENT KNOWLEDGE DISCOVERY

The number of tRNA genes compiled has already become huge and will undoubtedly increase more rapidly in the near future. For efficient knowledge discovery from such big data, analysis tools presently available are inadequate, and new tools suitable for big data analyses should be urgently required. Our group has developed a bioinformatics tool “BLSOM (Batch-Learning Self-Organizing-Map)” with oligonucleotide compositions (Abe et al., 2003), which can analyze more than one million genomic sequences simultaneously and allows us to efficiently acquire a wide range of knowledge from big sequence data. Oligonucleotides, such as penta- and heptanucleotides, often represent motif sequences responsible for sequence-specific protein binding (e.g., transcription factor binding). Occurrences of such motif oligonucleotides should differ from occurrences expected from the mononucleotide composition in each genome and may differ among genomic portions within a single genome. Actually, we have recently found that a pentanucleotides BLSOM for the human genome can detect characteristic enrichment of many transcription-factor-binding motifs in pericentric heterochromatin regions (Iwasaki et al., 2013a), showing that BLSOM can effectively detect the characteristic and combinatorial occurrences of functional motif oligonucleotides in the genomic sequence. Each tRNA has the characteristic and combinatorial occurrences of motif oligonucleotides, which are required to fulfill its function (e.g., binding to proper enzymes) and structural formation (e.g., clover leaf). To examine the usefulness of BLSOM for knowledge discovery from a huge amount of tRNAs, we have recently constructed a BLSOM with the pentanucleotide composition in all bacterial tRNAs in tRNADB-CE. Interestingly, tRNAs are separated primarily according to amino acid (should be published elsewhere), showing that the BLSOM can detect the characteristic combinations of motif oligonucleotides required for proper recognition by various enzymes, including aminoacyl-tRNA synthetases. Bacterial tRNAs with the same anticodon form their own territories, indicating this BLSOM can be used for an informatics method for assigning reliable tRNAs. For creating a high quality database, it is important to find errors that are slipped into the database, including those attributable to sequencing errors. An orphan tRNA located apart from the proper anticodon territories on BLSOM may be a candidate for erroneous cases, for which manual checks by experts have to be conducted. This type of new informatics strategies is required to find errors present in the huge amount of predicted tRNAs, especially in those predicted concordantly by all three programs and thus included in the database with no manual check. More importantly, BLSOM is an unsupervised clustering method with a strong visualization power (Abe et al., 2003) and this unsupervised data-mining method can be used for efficient knowledge discoveries from big sequence data (Iwasaki et al., 2013b). Informatics tools suitable for big data analyses have to be introduced to extract a wide range of knowledge from large-scale databases in the era of big data.

## Conflict of Interest Statement

The authors declare that the research was conducted in the absence of any commercial or financial relationships that could be construed as a potential conflict of interest.

## Footnotes

1 http://trnadb.bioinf.uni-leipzig.de/

2 http://lowelab.ucsc.edu/GtRNAdb/

3 http://mods.rna.albany.edu/home

4 http://modomics.genesilico.pl/

5 http://mamit-trna.u-strasbg.fr/

6 http://splits.iab.keio.ac.jp/splitsdb/

7 http://gobase.bcm.umontreal.ca/

8 http://www.ncbi.nlm.nih.gov/Traces/sra/

9 http://splits.iab.keio.ac.jp/splitsdb/

10 http://www.ncbi.nlm.nih.gov/sra
